# Evidence of HLA-DQB1 Contribution to Susceptibility of Dengue Serotype 3 in Dengue Patients in Southern Brazil

**DOI:** 10.1155/2014/968262

**Published:** 2014-04-10

**Authors:** Daniela Maria Cardozo, Ricardo Alberto Moliterno, Ana Maria Sell, Gláucia Andréia Soares Guelsin, Leticia Maria Beltrame, Samaia Laface Clementino, Pamela Guimarães Reis, Hugo Vicentin Alves, Priscila Saamara Mazini, Jeane Eliete Laguila Visentainer

**Affiliations:** Laboratório de Imunogenética, Departamento de Ciências Básicas da Saúde, Universidade Estadual de Maringá, Av. Colombo 5790, 87020-900 Maringá, PR, Brazil

## Abstract

Dengue infection (DI) transmitted by arthropod vectors is the viral disease with the highest incidence throughout the world, an estimated 300 million cases per year. In addition to environmental factors, genetic factors may also influence the manifestation of the disease; as even in endemic areas, only a small proportion of people develop the most serious form. Immune-response gene polymorphisms may be associated with the development of cases of DI. The aim of this study was to determine allele frequencies in the HLA-A, B, C, DRB1, DQA1, and DQB1 loci in a Southern Brazil population with dengue virus serotype 3, confirmed by the ELISA serological method, and a control group. The identification of the HLA alleles was carried out using the SSO genotyping PCR program (One Lambda), based on Luminex technology. In conclusion, this study suggests that DQB1∗06:11 allele could act as susceptible factors to dengue virus serotype 3, while HLA-DRB1∗11 and DQA1∗05:01 could act as resistance factors.

## 1. Introduction

Dengue infection (DI) is a resurging mosquito-borne disease that is often contracted in US travelers to Latin America, Asia, and the Caribbean. The clinical symptoms range from a simple febrile illness to hemorrhagic fever or shock [1]. The majority of the infected people remain asymptomatic; however, the majority of those who display symptoms develop a milder form of disease, dengue fever (DF), while others progress to severe forms of the disease, namely, dengue hemorrhagic fever (DHF) and dengue shock syndrome (DSS) [2].

Nowadays, there are currently four known serotypes: DEN 1, 2, 3, and 4, which are strongly related. The viruses belong to the genus flavivirus, family* Flaviviridae,* and in prevalent tropical and subtropical regions around the world, predominantly in urban and semiurban areas [3].

In Brazil the number of cases registered in first semester to 2013 was 204 million cases of dengue infections (DI), with the most part on North and Southeast region, and have been found to be serotypes 1, 2, and 3 [4].

In 2007, one of the states with the highest number of confirmed cases of DI serotype-3 was Paraná, with approximately 50,000 notified cases and 25,998 confirmed. One of the municipalities most affected was that of Maringá, with around 5,700 confirmed cases, which was considered the eighth highest DI incidence rate in the country.

The pathophysiology of DI and factors that results in severe clinical disease is poorly understood. Almost 50% of the infections are recognized to be clinically silent infections and in about 5–30% of the cases, the disease can be severe and complicated, with the symptoms of thrombocytopenia, plasma leakage, bleeding, and hypovolemic shock commonly referred to as DHF and DSS. The phenomenon of Antibody Dependent Enhancement (ADE) theory postulates that the infection with one dengue serotype during primary infection confers future protective immunity against that particular serotype but not with other serotypes during a secondary infection [5].

Besides them, HLA class I and II have been associated with primary DI and its several forms around the world (reviewed by Lan and Hirayama). The host HLA allele profile influenced the reactivity of DI-specific T cells and may be responsible for the immunopathology of DI [6–8].

However, genetic factors appear to be important in the manifestation of DI as, even in endemic areas, only a small proportion of people develop the DI or the most serious forms of the disease. During infection by the DI virus, a series of genes have their regulation mechanisms modified, among them, genes linked to high production of IFN-*γ*, as well as MIP-1*β*, RANTES, MBL2, IL-8, and IL-10 [9, 10].

HLA is encoded by the major histocompatibility complex, located on the chromosome 6 in humans. The genes encoding HLA class I (HLA-A, -B, and -C) and class II (HLA-DR, DQ, and DP) are the most polymorphic in the human genome [11].

Some studies showed positive associations, while others related negative associations between classical DF and HLA classes I and II alleles. In Cuba, HLA-B*35, DRB1*04, *07, *11, and DQB1*03:02 alleles were associated with protection against classical DF [12, 13]. Meanwhile in Thailand and Cuba the HLA-A*02:03, *31, B*15, *51, *52, DQB1*01, and *02:02 alleles have been associated with susceptibility to classical disease [13, 14].

### 1.1. Theory

In a Southern Brazilian population, our group has already found a strong association between HLA-DQ1 variant and DI, during an epidemic that occurred in 1995, characterized by the presence of DI virus serotype 1; however an association of DI with HLA class I antigens was not found [15].

For extending our studies to new cases of DI caused by the serotype 3 of virus, we conducted this case-control study, using more modern genotyping methodologies to characterize HLA classes I and II in patients from Southern Brazil.

### 1.2. Goal

The aim of this study was to determine allele frequencies in the HLA-A, B, C, DRB1, DQA1, and DQB1 loci in a Southern Brazil population with DI virus serotype 3, confirmed by the ELISA serological method, and a control group. The population DI is all dengue infections patients infected with dengue virus serotype 3, including classical DF, DHF, and DSS, according to World Health Organization (WHO).

## 2. Material and Methods

The institutional ethical review committees of the Universidade Estadual de Maringá in Brazil approved this study, and we obtained informed consent from the subjects upon enrollment.

### 2.1. Subjects

Ninety five patients with clinical features suggestive of DI who admitted to a general medical ward in at Health Center in Maringá, Southern Brazil, participated in the study. The quantitative detection of IgM and IgG antibodies to the DI antigen was performed by serology tests (ELISA-IgM and IgG Capture Kit, Brisbane, Australia), and the serotype-3 virus was characterized by cultures at the Paraná Central Laboratory for Infectious Diseases.

In addition, one hundred and seventy-three people did not present the symptoms and did not manifest the disease, from the same geographic area and age of patients participated as control cohort.

### 2.2. Obtaining Samples

After the separation of the serum for the serological tests, the coagulated whole-blood samples, collected without anticoagulant, were frozen at −20°C in Falcon-type tubes.

### 2.3. Dengue Serology

The diagnosis of a dengue virus infection in the patient groups was made by detection of IgM antibodies specific to the virus in serum samples with the Dengue IgM-Capture ELISA commercial kit (PanBio, Brisbane, Australia), using the immunoenzymatic ELISA (enzyme-linked immunosorbent assay) method. This test was performed during the period of the collections in 2007. The reading of the microplates was carried out in semiautomated equipment (ASYS Expert Plus, Cambridge, UK).

### 2.4. DNA Extraction and HLA Genotyping

The DNA extraction from blood samples used the NeoScience kit (One Lambda Inc., San Diego, CA, USA) or the salting out technique described for John et al. and modified by Cardozo et al. [16, 17].

HLA classes I and II genotypes of the patients and controls were analyzed by the reverse PCR-SSO (polymerase chain reaction, specific sequence of oligonucleotides) technique (One Lambda Inc., Canoga Park, CA, USA) with Luminex technology.

### 2.5. Calculation

The gene and genotypic frequencies were obtained by direct count, and the Hardy-Weinberg equilibrium was determined through the Arlequin software, version 3.1, available at: http://cmpg.unibe.ch/software/arlequin3/.

Patients and control individuals were then compared with regard to these frequencies by Fisher's Exact test (*P* < 0.05 was considered statistically significant), available at: http://www.graphpad.com/quickcalcs/contingency1.cfm. Bonferroni's correction for multiple tests was performed, and the risk of the development of DI in individual carriers with HLA alleles was calculated by OR (odds ratio) with a 95% confidence interval.

## 3. Results

95 presented clinical symptoms of DI and a positive confirmation of the disease for serotype 3 by ELISA serological exams. This group consisted of 50 (52.6%) females and 45 (47.4%) males, aged between 6 and 67 years (33.9 ± 14.8), with 85.4% being Caucasian, 10.4% Mulatto, and 4.2% Black.

The control group was made up of 173 individuals, with 94 (54.3%) being female and 79 (45.7%) male, aged between 15 and 72 years (29.3 ± 13.7), with 87.3% being Caucasian, 10.4% Mulatto, and 2.3% Black.

Difference statistically significant was not found on distributions to sex, age, and race when patient and controls were compared.

### 3.1. Differences in Classes I and II HLA Allele Frequencies between Individuals with Positive Serology for Dengue Infections and Controls

Tables 1 and 2 present the allele frequencies of HLA classes I and II, respectively, which are in Hardy-Weinberg equilibrium for this population.

When comparing the HLA frequencies of the two groups, several observed frequencies were significantly different from the expectations. HLA-C*07, DRB1*11, and DQA1*05:01 were negatively associated, while DRB1*15 and DQB1*06:11 were positively associated with DI. After using Bonferroni's correction, only* P* value for DQB1*06:11 (OR = 46.1; 95% IC = 2.7–785.3) keeps statistical significance.

In this present study, HLA-DRB1*15 and DQB1*06 alleles were in linkage disequilibrium in both groups of patients and controls (Δ′ = 0.92, *P* = 0.02), and the HLA-DRB1*15/DQB1*06:11 haplotype was associated with DF (21.0% versus 10.0%, *P* = 0.0269, OR = 2.30, 95% CI = 1.15–4.60).

Meanwhile, for HLA-DRB1*11 and DQB1*03 the linkage disequilibrium occurred negatively (Δ′ = 0.85, *P* = 0.85), and the haplotype HLA-DRB1*11/DQB1*03 was associated with protection against that with DI (15.8% versus 25.2%, OR = 0.5, 95% CI = 0.26–0.96, *P* = 0.0353).

The high resolution for allele definition to HLA-DRB1*11 and HLA-DRB1*15 variants was performed but the results were not clinically significant (data not shown).

There is no significant association found in other haplotypes (data no show).

## 4. Discussion

Brazil is a tropical country that has suffered the largest DI epidemics in the last five years. With a large genetic variability of European Caucasians, native Amerindians, and African blacks, who are distinct from a genetic point of view, in each region, mainly in terms of HLA allele polymorphism [18, 19].

In the light of this, the present study evaluated a population from the South of Brazil, one of the regions that experienced the largest dengue virus serotype 3 epidemic in 2007, with 5,700 confirmed cases, making it the municipality with the eighth highest DI incidence rate in the country (http://200.189.113.52/boletimdengue01.pdf).

The aim of this study was to verify a possible association between HLA classes I and II alleles and the epidemic that occurred at that time, using patients exhibiting DI-like symptoms unrelated to a previous epidemic.

In this study, we found negative and positive associations between HLA alleles and the serotype 3 DI. HLA-C*07, DRB1*11, and DQA1*05:01 were considered possible resistance factors to the disease, while HLA-DRB1*15 and DQB1*06:11 were considered susceptive factors.

Our group has already reported a positive association between HLA-DQ1 antigen (equivalent serologic to DQB1*05 and DQB1*06 alleles) and DF serotype 1 (57.7% versus 76.6%; *P* = 0.0052; *P*_*c*_ = 0.0262), in a study carried out by on individuals from the same geographic region. This fact suggests that both serotypes of virus responsible for classical DF may be positively associated with HLA-DQ1 variant in our region [15].

According to Nishimura et al. 1991, the particular alleles of the HLA-DQ locus may control the low immune response to natural antigens by a dominant genetic trait through the immune suppression mediated by CD8+ suppressor T cells. This phenomenon has been observed in several association studies involving DQ alleles and susceptibility to virus infections, like HIV, HPV, and HCV [20–23].

In this study, the HLA-DRB1*15/DQB1*06:11 haplotype was associated with DI. HLA-DRB1*15 and DQB1*06 alleles are in linkage disequilibrium in several populations (http://www.allelefrequencies.net/), suggesting that they may be together inherited.

However, Alagarasu et al. in India showed that the frequency of HLA-DRB1*07/15 genotype was significantly higher in DHF cases as compared to health controls and dengue fever patients in India [24].

In this study, a negative association of DRB1*11 and DQA1*05:01 was observed with DI. Falcón-Lezama et al. 2009 have shown an association of protection by HLA-DQB1*03:02 and susceptibility by HLA-DQB1*02:02 for classical DF, in a Mexican Mestizo population. DRB1*11 is in linkage disequilibrium with HLA-DQB1*03 in some populations, including in our population studied [25].

A limitation in our work is the relatively small number of samples collected; therefore, it seems that a large multicentric study with patients presenting the 4 serotypes of dengue virus, covering different areas in Brazil, could be important in this time of great epidemics.

## 5. Conclusion

In conclusion, although the strength of the majority of reported associations does not allow attributing a determining role on DI development, this study supports the possibility that host genetic factors indeed influence the development of the disease. DRB1*15 and DQB1*06:11 could act as susceptible factors to DI, while HLA-DRB1*11 and DQA1*05:01 alleles may act as resistance factors against DI caused by serotype 3.

Furthermore, we may emphasise the association between HLA-DQB1*06:11 and DI, significant even after the Bonferroni's correction, which confirms previous conclusions that HLA-DQ could influence DI infection in Southern Brazil.

## Figures and Tables

**Table 1:** Comparison of the frequencies of class I HLA alleles between the control group and patients with clinical-serologic diagnoses of dengue infection in the South of Brazil, occurring in 2007 during a serotype 3 virus epidemic.

HLA	Patients (95)		Controls (173)	
	*n*	%	*n*	%
A*01	12	6.3	38	11.0
A*02	44	23.2	98	28.3
A*03	27	14.2	30	8.7
A*11	10	5.3	16	4.6
A*23	12	6.3	16	4.6
A*24	17	8.9	33	9.5
A*25	6	3.2	3	0.9
A*26	7	3.7	12	3.5
A*29	11	5.8	13	3.8
A*30	8	4.2	20	5.8
A*31	5	2.6	13	3.8
A*32	7	3.7	8	2.3
A*33	5	2.6	9	2.6
A*34	0	0.0	1	0.3
A*36	3	1.6	1	0.3
A*66	2	1.1	1	0.3
A*68	9	4.7	29	8.4
A*74	5	2.6	5	1.4

B*07	32	4.7	30	6.9
B*08	20	2.1	14	4.6
B*13	10	2.6	7	2.0
B*14	16	5.3	13	4.3
B*15	45	9.5	32	8.1
B*18	26	5.8	20	6.9
B*27	10	2.1	4	2.0
B*35	63	12.1	37	14.2
B*37	4	1.1	4	0.6
B*38	15	3.2	15	3.8
B*39	13	3.2	13	3.5
B*40	14	3.2	21	6.6
B*41	10	1.6	6	0.0
B*42	7	1.6	3	0.6
B*44	49	10.5	46	7.8
B*45	7	0.5	2	1.4
B*46	0	0.0	0	0.3
B*48	3	1.6	2	0.0
B*49	9	1.1	8	3.5
B*50	10	2.6	9	2.0
B*51	39	8.4	29	11.8
B*52	10	3.2	2	1.2
B*53	14	3.2	10	1.4
B*54	0	0.0	0	0.3
B*55	5	1.6	5	2.0
B*56	2	1.1	2	0.3
B*57	15	3.2	5	1.4
B*58	13	3.7	10	2.0
B*81	4	0.5	0	0.3
B*82	2	1.1	1	0.0

C*01	12	4	6	2.9
C*02	38	21	19	6.1
C*03	41	19	34	11.6
C*04	86	35	55	17.1
C*05	20	7	24	4.0
C*06	46	18	27	7.2
C*07^a^	93	28	86	22.5
C*08	19	10	20	4.9
C*12	41	21	24	8.4
C*14	9	2	10	3.5
C*15	23	9	19	4.0
C*16	20	8	22	6.9
C*17	14	5	5	0.6
C*18	6	3	1	0.3

*n*: allelic number. ^a^ *P* = 0.037, OR = 0.59 (CI 95% = 0.37–0.95).

**Table 2:** Comparison of the frequencies of class II HLA alleles between the control group and patients with clinical-serologic diagnoses of dengue infection in the South of Brazil, occurring in 2007 during a serotype 3 virus epidemic.

HLA	Patients (95)		Controls (173)	
	*n*	%	*n*	%
DRB1*01	23	12.1	38	11.0
DRB1*03	20	10.5	40	11.6
DRB1*04	25	13.2	33	9.5
DRB1*07	23	12.1	38	11.0
DRB1*08	8	4.2	20	5.8
DRB1*09	7	3.7	4	1.2
DRB1*10	4	2.1	8	2.3
DRB1*11^a^	16	8.4	56	16.2
DRB1*12	3	1.6	5	1.4
DRB1*13	21	11.1	49	14.2
DRB1*14	9	4.7	24	6.9
DRB1*15^b^	21	11.1	19	5.5
DRB1*16	10	5.3	12	3.5

HLA	Patients (95)		Controls (173)	
	*n*	%	*n*	%

HLA-DQA*01:01	38	20.0	65	18.8
HLA-DQA*01:02	37	19.5	51	14.7
HLA-DQA*01:03	11	5.8	23	6.6
HLA-DQA*01:06	0	0.0	2	0.6
HLA-DQA*02:01	24	12.6	35	10.1
HLA-DQA*04:01	31	16.3	39	11.3
HLA-DQA*05:01^c^	8	4.2	21	6.1
HLA-DQA*05:10	40	21.1	107	30.9
HLA-DQA*06:01	0	0.0	1	0.3

HLA	Patients (96)		Controls (173)	
	*n*	%	*n*	%

HLA-DQB1*02:01	19	9.9	37	10.7
HLA-DQB1*02:02	26	13.5	34	9.8
HLA-DQB1*02:03	1	0.5	1	0.3
HLA-DQB1*03:01	30	15.6	73	21.1
HLA-DQB1*03:02	19	9.9	26	7.5
HLA-DQB1*03:03	3	1.6	5	1.4
HLA-DQB1*03:04	1	0.5	0	0.0
HLA-DQB1*03:05	1	0.5	1	0.3
HLA-DQB1*03:19	0	0.0	4	1.2
HLA-DQB1*03:22	0	0.0	1	0.3
HLA-DQB1*03:26	1	0.5	0	0.0
HLA-DQB1*04:01	0	0.0	3	0.9
HLA-DQB1*04:02	6	3.1	20	5.8
HLA-DQB1*05:01	31	16.1	50	14.5
HLA-DQB1*05:02	4	2.1	10	2.9
HLA-DQB1*05:03	12	6.3	20	5.8
HLA-DQB1*05:05	1	0.5	0	0.0
HLA-DQB1*06:01	20	10.4	34	9.8
HLA-DQB1*06:02	4	2.1	15	4.3
HLA-DQB1*06:03	1	0.5	8	2.3
HLA-DQB1*06:09	0	0.0	4	1.2
HLA-DQB1*06:11^d^	12	6.3	0	0.0

*n*: allelic number. ^a^ *P* = 0.014, OR = 0.48 (CI 95% = 0.27–0.86); ^b^ *P* = 0.03269, OR = 2.14 (CI 95% = 1.12–4.09); ^c^ *P* = 0.018, OR = 0.60 (CI 95% = 0.39–0.90); ^d^ *P* < 0.0001, OR = 46.13 (CI 95% = 2.71–785.3), *P*_*c*_ = 0.0044.

## Abbreviations

DI: Dengue infection
DF: Dengue fever
HLA: Human leukocyte antigen
PCR-SSO: Polymerase chain reaction, specific sequence of oligonucleotides.
